# Adjunct Therapy for CD4^+^ T-Cell Recovery, Inflammation and Immune Activation in People Living With HIV: A Systematic Review and Meta-Analysis

**DOI:** 10.3389/fimmu.2021.632119

**Published:** 2021-02-17

**Authors:** Yang Zhang, Taiyi Jiang, Aixin Li, Zhen Li, Jianhua Hou, Meixia Gao, Xiaojie Huang, Bin Su, Hao Wu, Tong Zhang, Wei Jiang

**Affiliations:** ^1^Center for Infectious Diseases, Beijing YouAn Hospital, Capital Medical University, Beijing, China; ^2^Beijing Key Laboratory of AIDS Research, Beijing, China; ^3^Department of Microbiology and Immunology, Medical University of South Carolina, Charleston, SC, United States; ^4^Division of Infectious Diseases, Department of Medicine, Medical University of South Carolina, Charleston, SC, United States

**Keywords:** CD4^+^ T-cell, immune activation, inflammation, immunologic non-responder, people living with HIV, adjunct therapy

## Abstract

**Background:** HIV infection results in immune homeostasis perturbations, which is characterized by CD4^+^ T-cell depletion, immune activation, and inflammation. Effective antiretroviral therapy (ART) does not fully restore immunologic and clinical health in people living with HIV (PLWH). Various drugs have been used to improve their immune status and CD4^+^ T-cell counts, but no measures have been tested effective. Here we conduct a systematic review and meta-analysis of existing clinical studies on improving CD4^+^ T-cell count while decreasing inflammation and immune activation.

**Methods:** We retrieved possible relevant publications from a total of five electronic databases and selected eligible studies, which dealt with outcomes of medical therapy for CD4^+^ T-cell count recovery, inflammation, and immune activation with or without ART. We paid particular attention to immunologic non-responders with a favorable treatment regimen.

**Results:** Thirty-three articles were included in the systematic review and meta-analysis. However, there were no safe and effective medications specific for improving CD4^+^ T-cell reconstitution. The immunological benefits or adverse events mainly depend on the safety, dosage, and duration of the candidate medication use, as well as whether it is combined with ART.

**Conclusion:** Under the “safe, combined, adequate and long (SCAL)” principles, alternative approaches are needed to accelerate the recovery of CD4^+^ T-cells, and to prevent adverse long-term outcomes in PLWH with standard ART treatment.

## Introduction

Since 1996, the morbidity and mortality of HIV-related diseases were dramatically declined by the wide use of antiretroviral therapy (ART) (1). ART is a standardized combination of several anti-HIV drugs (1), being classified as etiological therapy. Theoretically, treatment-experienced people living with HIV (PLWH) persistently suppress the HIV viral load, and their CD4^+^ T-cell counts increase gradually. However, PLWH fail to achieve normalization of CD4^+^ T-cell counts despite persistent blood virological suppression, especially in immunological non-responder (INR, usually defined as PLWH under ART with viremia and CD4^+^ T-cell counts < 350/μl in the blood) showing severe immunological dysfunction (2). This immunological dysfunction involves chronic immune activation and systemic inflammation (3), and is thought to contribute not only to HIV disease progression, but also to mortality and emerging non-AIDS morbidity (4, 5). To date, there is still a lack of effective adjunct medical therapy to further enhance CD4^+^ T-cell counts for PLWH, including INRs.

For over two decades, researchers have tested multiple medication candidates to recover CD4^+^ T-cell count. Montaner et al. have assessed the effect of hydroxyurea in PLWH in 1997; However, the CD4^+^ T-cell counts did not change significantly; or even significantly decreased during the washout phase (6). Because the level of immune activation and inflammation were independently associated with the subsequent rate of CD4^+^ T-cell losses (7, 8), the immunomodulators or immunosuppressants such as glucocorticoid (9) and cyclosporine (10), may be beneficial to PLWH or INRs. In recent years, PLWH may have more benefits by “early treatment” (11), and they will not go through the process from immunological destruction to reconstruction, which means that INRs may be fewer and fewer. However, although their CD4^+^ T-cell count was high, it did not reach normalization (1). Besides, ART does not eliminate inflammation and immune activation (4, 5), so researchers have paid more attention to other traditionally non-AIDS-related morbidities (12, 13). In other words, hypertension, hyperglycemia, and hyperlipidemia may be similar to the low CD4^+^ T-cell count, which may be the adverse consequences of inflammation and immune activation. Besides, some candidate drugs also show anti-inflammatory and inhibitory effects on excessive immune activation in the general population. Take rosuvastatin (14) and sitagliptin (15) as examples, scientists hypothesized that the CD4^+^ T-cell recovery over time could be explained by the improved use of non-HIV-specific preventive interventions. Unfortunately, even though some candidates could reduce immune activation or chronic inflammation to varying degrees, they cannot directly increase CD4^+^ T-cell count recovery, and the mechanisms underlying these candidates are far from known.

Therefore, we searched and summarized the literature on the case-control study of medication-assisted treatment of CD4^+^ T-cell count recovery. The second aim was to summarize biomarkers of inflammation and immune activation in PLWH. An intervention that decreased immune activation or inflammation or both in PLWH might therefore be beneficial. Besides, for CD4^+^ T-cell count recovery in INRs, we separately analyzed articles related to incomplete immune reconstitution in PLWHs. We hope to put forward the therapeutic principle of adjunct therapy for CD4^+^ T-cell recovery, and provide a basis for further searching for an inexpensive, safe, and well-tolerated candidate intervention.

## Methods

The protocol in this study has been deposited and registered in the PROSPERO database (CRD42020210393).

### Participants

Adult PLWH with or without ART.

### Interventions

Any auxiliary medicine treatment in junction with or without ART, but not including inaccurate dose or non-drug treatment studies (e.g., immunotherapy and cell therapy).

### Comparators

PLWH with or without ART + adjunct treatment vs. PLWH with or without ART (placebo or blank control).

### Search Strategy

Searches were limited to data published before February 29, 2020 using a combination of population-related terms (HIV OR AIDS), immunology-related terms (“immune reconstitution” OR “CD4^+^ T-cell recovery”), and treatment-related terms (treatment OR therapy). All searches were limited to peer-reviewed journal articles in English.

### Data Sources

Comprehensive searches were conducted in PubMed, Web of Science, and Cochrane library. Besides, Google Scholar and GeenMedical were used as supplementary sources.

### Studies Sections and Data Extraction

We included [1] studies conducted in PLWH; [2] any auxiliary medicine treatment in junction with or without ART treatment; [3] studies with at least two groups (adjunct therapy group vs. control group) including randomized controlled trials (RCT) and non-RCTs; (4) the results of the research which should include the CD4^+^ T-cell count; and (5) articles were written in English and excluded those studies without peer-review process, case reports, theoretic studies, conference abstracts and samples overlapped with the other included studies. We also excluded studies that did not include accurate doses (e.g., probiotics/prebiotics, fish oil, and chocolate) and non-drug treatment studies (e.g., immunotherapy and cell therapy). Two authors (Yang Zhang and Taiyi Jiang) used an Excel spreadsheet to independently screen for eligibility.

Two authors (Yang Zhang and Jianhua Hou) used an Excel spreadsheet to independently extract data from each study. The opinions on the included data were resolved by consensus after consulting a 3rd reviewer (Aixin Li). The summary statistics of each outcome were means, standard deviations (SD), and the number of participants. We also coded the mean change and SD of mean change if necessary. Related information from other studies was coded for use as well.

### Data Analysis

We considered using meta-analytical methods when three or more studies with the same treatment, or systematic review was used to summarize the results.

For the meta-analysis, random-effect models were adopted to estimate the pooled estimation of effect sizes. The variation in effect sizes across studies was assessed by the Cochrane Q with a threshold of *P* < 0.1. The *I*^2^ statistic was calculated to estimate the proportion of true heterogeneity in observed variance. The funnel plot and Egger's regression intercept test were used to estimate publication bias. If the *P*-value for Egger's test is larger than 0.1, publication bias exists, and we should draw cautious conclusions. Besides, we calculated Egger's test only if three or more comparisons are included in specific outcomes.

For the systematic review, we grouped our results classified by types of treatment. The outcomes of interest were changes in CD4^+^ T-cell count, viral load, other immune system biomarkers such as inflammation or immune activation, and medical side effects.

## Results

### Study Selection and Characteristics

**Figure 1:**
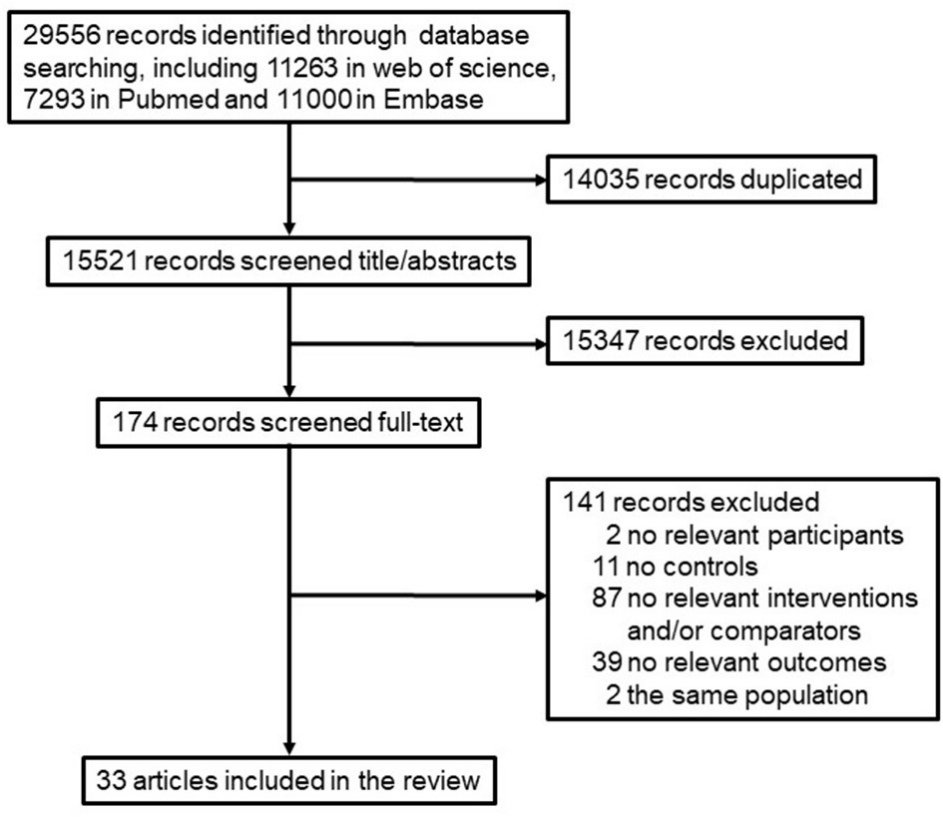
A flow diagram of the studies retrieved for the review.

Our literature searches returned 29,556 records, including 11,263 in the web of science, 7,293 in Pubmed, and 11,000 in Embase. After duplicates removal, 15,521 studies were remained. Of these, 14,447 were excluded during the title and abstract screening, leaving 174 publications for full-text evaluation. Of these, 141 were excluded for various reasons after full-text review, as detailed in the flow chart. Thirty-three studies were included in the final review (Figure 1, Table 1).

**Table 1:** Characteristic of included articles.

**First author**	**Publication year**	**Design**	**Number of participants**	**ART**	**Adjunct therapy**	**Drug usage**	**Duration of medication use**	**Relevant outcome measures**
Rusconi (18)	2010	RCT	47 vs. 50^a^	Yes	Maraviroc	300 mg, twice daily	48 weeks	CD4^+^ T-cell count, IL-7, CD38, and HLA-DR
Hunt (16)	2013	RCT	23 vs. 22^a^	Yes	Maraviroc	300 mg, twice daily	24 weeks	CD4^+^ T-cell count, soluble CD14, LPS, CD38, and HLA-DR
Minami (17)	2017	Observational research	18 vs. 14^a^	Yes	Maraviroc	300 mg, twice daily	24 weeks	CD4^+^ T-cell count, IFN-γ
McComsey (9)	2001	RCT	20 vs. 21^a^	Yes^b^	Prednisone	0.5 mg/kg, once daily	8 weeks	CD4^+^ T-cell count, IL-6 TNF-α, and CD38
Kasang (19)	2012	Observational research	27 vs. 31 vs. 30 vs. 13^c^	Yes/No	Prednisolone	5 mg, once daily	N.A.	CD4^+^ T-cell count, soluble CD14, LPS binding protein, suPAR, soluble CD40L, and CD38
Kasang (20)	2016	RCT	13 vs. 24	No	Prednisolone	5 mg, once daily	2 years	CD4^+^ T-cell count, soluble CD14, suPAR, CD38, and HLA-DR
Rizzardi (10)	2002	Non-RCT	9 vs. 29	Yes	Cyclosporine	0.3~0.6 mg/kg, twice daily	8 weeks	CD4^+^ T-cell count and IFN-γ
Lederman (21)	2006	RCT	22 vs. 20	Yes	Cyclosporine	4 mg/kg, twice daily	2 weeks	CD4^+^ T-cell count and CD38
Markowitz (22)	2010	RCT	28 vs. 13	Yes	Cyclosporine	0.3 mg/kg, once daily	4 weeks	CD4^+^ T-cell count, CD38 and HLA-DR
Murray (23)	2010	RCT	6 vs. 3 vs. 4^d^	No	Chloroquine	250 mg/500 mg, once daily	2 months	CD4^+^ T-cell count, LPS, CD38, and HLA-DR
Jacobson (24)	2016	RCT	16 vs. 17 vs. 18 vs. 19^e^	No	Chloroquine	250 mg, once daily	13 weeks	CD4^+^ T-cell count, CD38, and HLA-DR
Paton (7)	2012	RCT	42 vs. 41	No	Hydroxychloroquine	400 mg, once daily	48 weeks	CD4^+^ T-cell count, CD38, and HLA-DR
Funderburg (14)	2015	RCT	72 vs. 75	Yes	Rosuvastatin	10 mg, once daily	48 weeks	CD4^+^ T-cell count, soluble CD14, soluble CD163, IL-6, soluble receptors of TNF-α, IP-10, VCAM-1, soluble intercellular adhesion molecule 1, D-dimer, fibrinogen, CD38, and HLA-DR
Weijma (25)	2016	RCT	13 vs. 15	No	Rosuvastatin	20 mg, once daily	8 weeks	CD4^+^ T-cell count, neopterin, soluble TLR2, soluble TLR4, IL-6, IL-1Ra, IL-18, D-dimer, CRP, CD38, and HLA-DR
Nakanjako (26)	2015	RCT	15 vs. 15^a^	Yes	Atorvastatin	80 mg, once daily	12 weeks	CD4^+^ T-cell count, CD38, and HLA-DR
Best (27)	2015	RCT	18 vs. 20	Yes	Sitagliptin	100 mg, once daily	8 weeks	CRP, C-X-C motif chemokine 10, MCP-1, EGF-like modulecontaining, mucin-like hormone receptor 1
Dubé (15)	2019	RCT	42 vs. 42	Yes	Sitagliptin	100 mg, once daily	16 weeks	CD4^+^ T-cell count and soluble CD14
Hunt (29)	2011	RCT	14 vs. 16^a^	Yes	Valganciclovir	900 mg, once daily	8 weeks	CD4^+^ T-cell count, CD38, and HLA-DR
Yi (28)	2013	RCT	20 vs. 20 vs. 20^f^	Yes	Valacyclovir	500 mg/1 g, twice daily	12 weeks	CD4^+^ T-cell count
Missailidis (30)	2019	RCT	81 vs. 86	No	Vitamin D3 and phenylbutyrate	5000 IU vitamin D3 and 500 mg phenylbutyrate, once daily	16 weeks	CD4^+^ T-cell count, soluble CD14, antimicrobial peptide LL-37, kynurenine/tryptophan-ratio
Ashenafi (31)	2019	RCT	95 vs. 102	No	Vitamin D3 and phenylbutyrate	5000 IU vitamin D3 and 1,000 mg phenylbutyrate, once daily	16 weeks	CD4^+^ T-cell count
Tenorio (32)	2015	RCT	43 vs. 22^a^	Yes	Rifaximin	550 mg, twice daily	4 weeks	CD4^+^ T-cell count, soluble CD14, soluble CD163, IL-6, LPS, CRP, D-dimer, CD38, and HLA-DR
Lindboe (33)	2016	RCT	28 vs. 18	Yes	Recombinant human growth hormone	0.7 mg, once daily	40 weeks	CD4^+^ T-cell count, CRP, and suPAR
Srinivasa (34)	2018	RCT	25 vs. 21	Yes	Eplerenone	50 mg, once daily	6 months	CD4^+^ T-cell count, CRP, IL-6, PAI-1, MCP-1, and adiponectin
Bourke (35)	2019	RCT	144 vs. 149	Yes	Cotrimoxazole	^g^	48 weeks	CD4^+^ T-cell count, CRP, IL-6, soluble CD14, and TNF-α
Macatangay (36)	2020	RCT	17 vs. 18	Yes	Dipyridamole	100 mg, four times daily	12 weeks	CD4^+^ T-cell count, soluble CD163, soluble CD14, IL-6, CD38, and HLA-DR
Natsag (37)	2016	RCT	36 vs. 34	Yes	Alendronate	70 mg, once weekly	48 weeks	CD4^+^ T-cell count, IL-6 and soluble receptors for TNF-α
Somsouk (38)	2014	RCT	15 vs. 18^a^	Yes	Mesalamine	1,500 mg, once daily	12 weeks	CD4^+^ T-cell count, soluble CD14, IL-6, D-dimer, kynurenine to tryptophan ratio, CD38, and HLA-DR
O'Brien (39)	2017	RCT	38 vs. 38 vs. 36^h^	Yes	Aspirin	100 mg/300 mg, once daily	12 weeks	CD4^+^ T-cell count, soluble CD14, IL-6, soluble CD163, CD38, and HLA-DR
Gupta (40)	2013	RCT	13 vs. 13	Yes	Pentoxifylline	400 mg, 3 times daily	8 weeks	CD4^+^ T-cell count, CRP, IL-6, soluble TNF-α receptors, tissue inhibitor of metalloproteinase-1, MCP-1, IP-10, PAI-1, CD38, and HLA-DR
Vergara (41)	2017	RCT	16 vs. 14	No	Thalidomide	200 mg, once daily	3 weeks	CD4^+^ T-cell count, CRP, LPS, CD38 and HLA-DR
Hsue (42)	2019	RCT	86 vs. 90	Yes	Methotrexate	5~15 mg, once weekly	24 weeks	CD4^+^ T-cell count, CRP, IL-6, soluble CD163, soluble CD14, VCAM-1, IP-10, D-dimer and fibrinogen
Kent (43)	2018	RCT	34 vs. 31	Yes	Vorapaxar	2.5 mg, once daily	12 weeks	CD4^+^ T-cell count, D-dimer, CRP, IL-6, soluble CD14, and soluble CD163

ART, antiretroviral therapy; RCT, randomized controlled trial; IL, interleukin; LPS, lipopolysaccharide; IFN, interferon; TNF, tumor necrosis factor; N.A., not applicable; suPAR, soluble urokinase plasminogen activated receptor; IP-10, IFN-γ-inducible protein 10; VCAM-1, vascular cell adhesion molecule 1; TLR, Toll-like receptor; CRP, C-reactive protein; MCP-1, monocyte chemoattractant protein 1; PAI-1, plasminogen activator inhibitor 1;

a Participants were immunological non-responders;

b All one of the patients had been taking stable ART;

c 27 received prednisolone, 31 received prednisolone in combination with ART, 30 received HAART alone and 13 received neither ART nor prednisolone;

d 6 receiving 250 mg of chloroquine daily, 3 receiving 500 mg of chloroquine daily, and 4 receiving placebo;

e A RCT comparing chloroquine to placebo administration in two sequential cohorts of HIV-infected individuals: off-ART [cohort 1: arms A (N = 16) and B (N = 17)] and on-ART [cohort 2: arms C (N = 18) and D (N = 19)], then randomized 1:1 to receive chloroquine 250 mg for the first 12 weeks and then to cross over to placebo for 12 weeks (arms A and C) or placebo for 12 weeks then cross over to chloroquine 250 mg for 12 weeks (arms B and D);

f 20 received valacyclovir 500 mg twice daily, 20 received valacyclovir 1 g twice daily, or 20 received placebo;

g 200 mg of sulfamethoxazole/40 mg of trimethoprim, 400 mg of sulfamethoxazole/80 mg of trimethoprim, or 800 mg of sulfamethoxazole/160 mg of trimethoprim for body weight 5–15, 15–30, or >30 kg, respectively;

h *38 received daily aspirin 100 mg, 38 received aspirin 300 mg, or 36 received placebo*.

Among 33 studies, one study was a non-randomized intervention study, two were observational studies, and others were RCTs. Regarding ART, 30 studies used ART in combination with these auxiliary treatments. In addition, the visit period for all studies ranged from 3 to 48 weeks. The reported outcomes included CD4^+^ T-cell count, viral load, inflammation biomarkers, and medical side effects. The detailed information of each treatment was reviewed and summarized below.

### Synthesized Findings

#### Maraviroc

**Figure 2:**
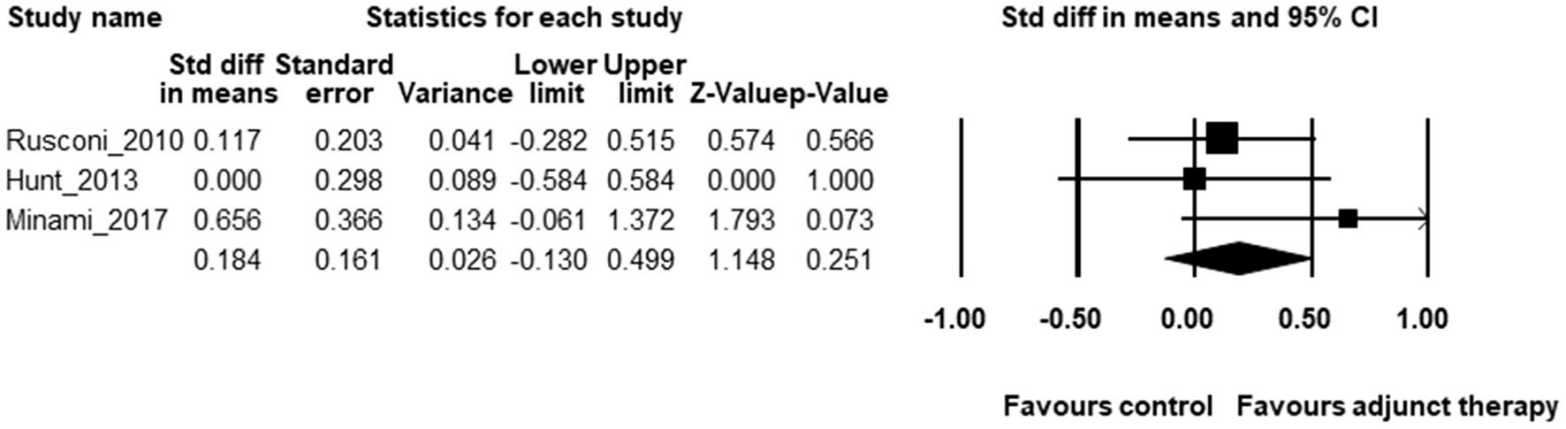
The forest plot for the effects of maraviroc on CD4^+^ T-cell counts.

The CC chemokine receptor type 5 inhibitor maraviroc has been hypothesized to decrease T-cell activation in PLWH (16), and associated with an enhanced CD4^+^ T-cell response independent of virological suppression (17). Two RCTs (16, 18) and one observational study (17) reported the effects of maraviroc on CD4^+^ T-cell counts. The pooled estimate of effects on CD4^+^ T-cell count was 0.184, 95% CI 0.13 ~ 0.499, *p* = 0.254, Figure 2. The heterogeneity across studies was not significant and small [*Q*(2) = 2.153, *p* = 0.341, *I*^2^ = 7.122%]. The publication bias was not significant (Intercept = 2.177, *p* = 0.57).

Unexpectedly, Hunt et al. found that compared with the placebo group, maraviroc-treated patients with ART experienced a tremendous median increase in CD38^+^HLA-DR^+^CD8^+^ T cells (16). Furthermore, Rusconi et al. also reported that PLWH accepted maraviroc showed higher levels of HLA-DR^+^CD38^+^CD8^+^ T cells as compared to those without maraviroc (18). These studies suggest that maraviroc does not effectively reduce immune activation, at least in INRs.

#### Glucocorticoid

McComsey et al. reported a double-blinded RCT of prednisone in 41 patients (40 with ART) with HIV-1 infection (9). They found that 8 weeks of prednisone administration is reasonably safe in advanced HIV-1 disease, and decreases immune activation (tumor necrosis factor α levels and percentages of CD38^+^CD8^+^ T cells). However, there was no effect on HIV-1 RNA levels or CD4^+^ T-cell count.

An observational study demonstrates that although PLWH without ART-treated with low-dose prednisolone display significantly lower general immune activation, prednisolone was not beneficial to CD4^+^ T-cell recovery and viral load controls (19). In double-blinded RCT, prednisolone could increase the odds of CD4^+^ T-cell count recovery as well as decrease immune activation and increase HIV viral load (20). These studies have shown that, without the escort of ART, the benefits of glucocorticoids on CD4^+^ T-cell reconstitution and the risk of viral activation may coexist.

#### Cyclosporine

An RCT evaluating the immune-modulating effects of combining cyclosporine treatment with ART during primary HIV-1 infection has shown that the net increase over baseline values in both CD4^+^ T-cell count and CD4/CD8 ratio was significantly greater in PLWH receiving cyclosporine in combination with ART than those receiving ART alone (10). However, two other cyclosporine RCTs have shown no significant differences between treatment arms in levels of CD4^+^ T-cell counts (21, 22). Interestingly, treatment with cyclosporin A (4 mg/kg, bid) for 2 weeks provided only a transient enhancement in circulating CD4^+^ T-cell restoration (21). However, adjunctive therapy with cyclosporine (0.3 mg/kg, qd) even for 4 weeks did not provide apparent virologic or immunologic benefits (22), suggesting that adequate doses of cyclosporine are needed to suppress immune activation.

#### Chloroquine and Hydroxychloroquine

Three RCTs have tested the effects of chloroquine and its derivatives on CD4^+^ T-cell counts in PLWH. One double-blind RCT indicated that treatment with chloroquine could reduce the systemic T-cell immune activation in participants without ART, but they did not report CD4^+^ T-cell counts and viral load (23). Another double-blind RCT in PLWH off and on ART demonstrated no significant differences in the changes of CD4^+^ T-cell counts between the chloroquine arm and the placebo arm in either cohort (24). However, chloroquine modestly reduced the proportions of CD8^+^T cells co-expressing CD38 and HLA-DR in ART-treated HIV-infected participants. In contrast, this effect on immune activation was not found in the off-ART group during chloroquine use. This study showed that even if chloroquine could not increase CD4^+^ T-cell count, it should be combined with ART to reduce immune activation in PLWH.

Moreover, a double-blind RCT performed in the United Kingdom indicated that among HIV-infected patients not taking ART, hydroxychloroquine compared with placebo did not reduce CD8^+^ T cell activation, but did significantly decrease CD4^+^ T-cell count and increase HIV-RNA replication (7). These studies indicated that adjuvant treatments with immune modulators to increase CD4^+^ T-cell count should consider the combination with ART. Otherwise, it would bring about the risk of increased HIV replication and even further the decline of CD4^+^ T-cell counts.

#### Rosuvastatin and Atorvastatin

Statins, or 3-hydroxy-3-methylglutaryl coenzyme A reductase inhibitors, have anti-inflammatory effects, licensed and widely marketed for the treatment of dyslipidemia (14). There are two RCTs on rosuvastatin and one RCT on atorvastatin involving statins in PLWH, which indicated that statins could not increase CD4^+^ T-cell counts in PLWH on ART. However, rosuvastatin may significantly reduce several inflammatory markers, and lymphocyte and monocyte activation in ART-treated subjects in a 48-week follow up (14) rather than 8-weeks (25). Besides, atorvastatin could also reduce the T-cell immune activation among ART-experienced adults in a 24-week study (26). These studies have shown that statins are needed for a long period to reduce immune activation and inflammation among PLWH.

#### Sitagliptin

Dipeptidyl peptidase-4 inhibitors (e.g., sitagliptin) are a relatively new class of oral antidiabetes medications. In animal models and clinical studies of type 2 diabetes, dipeptidyl peptidase-4 inhibitors appear to have many cardiometabolic, anti-inflammatory, and immunoregulatory benefits in addition to their glucoregulatory actions (15). Both 8 (27) and 16 weeks (15) of sitagliptin had pleiotropic anti-inflammatory and immune regulatory effects in PLWH during ART. However, there were no significant between-arm differences in CD4^+^ T-cell counts.

#### Valganciclovir and Valacyclovir

Herpes simplex virus type 2 is a common HIV coinfection, contributing to the increased systematic inflammation and immune activation despite suppressive ART (28). Either RCTs of anti-herpes simplex virus medication valganciclovir (29) or valacyclovir (28) could not increase CD4^+^ T-cell counts in PLWH. Still, valganciclovir-treated participants had a significantly greater reduction in CD8^+^ T-cell activation than those from the placebo group, which was not observed in the study of valacyclovir.

#### Vitamin D3 and Phenylbutyrate

Poor nutritional status is common among PLWH (30, 31). Vitamin D3 and phenylbutyrate possess pleiotropic immunomodulatory functions that could simultaneously prevent chronic immune activation and dysregulation (30, 31). Two double-blinded RCTs in treatment-naive HIV patients indicated that daily nutritional supplementation with Vitamin D3 and phenylbutyrate could not change the viral load, CD4^+^ T-cell counts, and levels of inflammation (30, 31).

#### Other Drug Candidates

Some candidates were conducted in one RCT, which was demonstrated the anti-inflammatory and immunomodulatory effects by rifaximin (32), recombinant human growth hormone (33), eplerenone (34), cotrimoxazole (35), and dipyridamole (36) decreased immune activation significantly among persons with HIV-1 infection receiving ART. Further studies are necessary to uncover the clinical potential of these candidates on CD4^+^ T-cell count recovery in PLWH.

However, HIV-associated changes of CD4^+^ T-cell counts and immune activation are not impacted by alendronate (37), mesalamine (38), and aspirin (39). More frustratingly, the pentoxifylline (40), thalidomide (41), methotrexate (42), and vorapaxar (43) had no benefits on immune activation, inflammation, and CD4^+^ T-cell count recovery in PLWH receiving ART but at risk of poor outcomes.

#### Adjuvant Therapy for Poor Immune Reconstitution

There are eight studies on INRs in the above research. For maraviroc, only one study proved that maraviroc could increase the CD4^+^ T-cell counts (17). One study reported that maraviroc could reduce inflammation (18), but another study showed that maraviroc increased immune activation (16). However, two studies showed that maraviroc has serious adverse events (17, 18).

For the other five candidates, which were only conducted in one study, prednisone (9), valganciclovir (29), atorvastatin (26), and rifaximin (32) have all been reported to reduce inflammation and immune activation to varying degrees, except for mesalamine (38). As expected, prednisone has been reported more adverse events (9).

## Discussion

### Summary of Main Findings

HIV is associated with increased systematic inflammation and immune activation that persist despite suppressive ART (44). The elevated immune activation and inflammation are associated with an increased risk of non-AIDS diseases and mortality among PLWH (12, 13). While ART reduces the level of inflammatory biomarkers, it does not result in normalization of CD4^+^ T-cell counts and host immunity (45). Interventions reducing immune activation request a deep understanding of pathogenesis and a balance thought of therapeutic benefit or side effects. In this systematic review, only maraviroc can be used for meta-analysis. However, maraviroc does not significantly increase the CD4^+^ T-cell counts of INR. Moreover, most of the included studies have shown that the maraviroc and other target drugs could reduce inflammation or immune activation based on different biomarkers. Besides, we have also evaluated the safety of candidates, especially in HIV load and CD4^+^ T-cell counts. In general, there is no well-established adjunct therapy to increase CD4^+^ T-cell counts.

In this systematic review and meta-analysis, the use of research drugs that bring immunological benefits in PLWH usually has the following characteristics, which may also be the principle of searching for ideal candidates in the future. First, this clinical study design should be based on the combined ART (i.e., principle “combined”). Hydroxychloroquine (7) and thalidomide (41) did not reduce inflammation and immune activation without ART, but led to a greater decline in CD4^+^ T-cell counts and an increase in viral replication. In contrast, even if other studies did not bring immunological or virological benefits, or even showed the adverse effects of the candidate drug, the candidates combined with ART would not lead to the poor outcomes such as a decrease in CD4^+^ T-cell counts or an increase in HIV RNA load.

Second, the preexisting immune system abnormalities in the setting of HIV infection may overpower the immunomodulatory effects of the overwhelming majority of candidates (3). Moreover, ART, low-level HIV replication, microbial translocation across damaged mucosal surfaces, and chronic coinfections may contribute to persistent inflammation during influential virologic ART (4, 5). Accordingly, an adequate dose for an extended period of candidate interventions is needed to moderate them (i.e., principle “adequate” and “long”). Two cyclosporine studies indicated that adequate cyclosporine doses (21) could suppress immune activation rather than fewer doses (22). Besides, insufficient use of a loading vorapaxar dose in PLWH may not achieve the general effect, as some cardiovascular studies have shown (43). Similarly, the 48-week treatment of rosuvastatin (13) may significantly reduce immune activation and inflammation among PLWH in the 8-week study (7).

Finally, immune activation and inflammation persist in most ART-suppressed PLWH (5). Therefore, reducing persistent immune activation has emerged as a major priority (4). Considering the principles of “combined, adequate and long,” as well as drug interaction with ART, evidently and most importantly, a “safe” intervention that suppresses immune activation and increases CD4^+^ T-cell counts would be attractive. Our review indicated that the vast majority of candidates above might not be studied further as a treatment to increase the CD4^+^ T-cell counts in PLWH. Therefore, we propose the four principles of adjunct therapy for CD4^+^ T-cell recovery in PLWH, namely the “SCAL” principles of safe, combined, adequate, and long. Maybe existing proven interventions in HIV-negative populations to modify inflammation and immune activation risks remain the best available methods to reduce non-AIDS disease in PLWH.

So how do we screen drug candidates to increase CD4^+^ T-cell counts of PLWH? Much has been learnt about candidates since assessing the effect on hydroxyurea in PLWH in 1997 (6). Although the exact proinflammatory mechanisms are unclear, interventions that reduce inflammation and immune activation are believed to reduce non-AIDS disease risk in PLWH (4, 5). Bioinformatics technology should be used to explore the potential differences in the immune system between PLWH and healthy controllers, especially in INRs. Next, chemoinformatics could be further used to match candidates that meet the “SCAL” principles, just like scientists' outstanding performance during the SARS-CoV-2 epidemic (46). Given the potential for complex and unpredictable effects, interventions for immune activation and CD4^+^ T-cell recovery must be evaluated rigorously and comprehensively in adequately powered randomized controlled trials. Also, in the context of fully suppressive ART, multifarious immunotherapeutic approaches such as vaccines or cytokines (47), dietary supplements (48), might further reduce immune activation, and improve CD4^+^ T-cell count in PLWH. However, because the intervention was difficult to standardize or the number of subjects was small, the safety and effectiveness of these methods need to be further verified.

## Limitations

The use of different biomarkers in this included literature above might miss biological effects with the potential for clinical importance. Still, it is much more efficient than devoting the resources for CD4^+^ T-cell count recovery as a clinical endpoint study. Besides, we focused on individuals with incomplete CD4^+^ T-cell count recovery. They tend to have the higher persistent immune activation levels and are at the highest risk for morbidity and mortality. However, despite a large number of candidates, few studies adopt the control group, so we didn't include these studies, which also excluded the effect of the slow increase in CD4^+^ T-cell count among PLWH or INR.

## Conclusions

To date, there are no safe and effective medications specific to improving CD4^+^ T-cell reconstitution. There are still considerable challenges in the adjuvant treatment of CD4^+^ T-cell count recovery, as well as the interventions of inflammation and immune activation. The immunological benefits or adverse events mainly depend on the safety, dosage, and duration of the candidate medication use, and whether it is combined with ART. Therefore, we propose the four principles of the “SCAL” principles. Under the guidance of “SCAL” principles, it is necessary to develop effective drugs and design rigorous clinical trials to verify in PLWH treated with standard ART in the future.

## Data Availability Statement

The original contributions presented in the study are included in the article/supplementary material, further inquiries can be directed to the corresponding author/s.

## Author Contributions

YZ, TJ, AL, HW, TZ, and WJ had full access to all of the data in the study and are responsible for the integrity of data and the accuracy of data analysis. YZ, HW, TZ, and WJ study concept and design. YZ, TJ, AL, JH, and MG acquisition, analysis, and interpretation of data. YZ, TJ, and AL drafting the manuscript. ZL, XH, BS, HW, TZ, and WJ critical revision of the manuscript for important intellectual content. YZ, ZL, XH, BS, HW, TZ, and WJ obtained funding. YZ, HW, TZ, and WJ study supervision. All of the authors gave the publishing approval.

## Conflict of Interest

The authors declare that the research was conducted in the absence of any commercial or financial relationships that could be construed as a potential conflict of interest.
